# Impact of nitrogen fertilization on soil–Atmosphere greenhouse gas exchanges in eucalypt plantations with different soil characteristics in southern China

**DOI:** 10.1371/journal.pone.0172142

**Published:** 2017-02-13

**Authors:** Kai Zhang, Hua Zheng, Falin Chen, Ruida Li, Miao Yang, Zhiyun Ouyang, Jun Lan, Xuewu Xiang

**Affiliations:** 1 State Key Laboratory of Urban and Regional Ecology, Research Center for Eco-Environmental Sciences, Chinese Academy of Sciences, Beijing, China; 2 College of Grassland and Environment Sciences, Xinjiang Agricultural University, Urumqi, Xinjiang, China; 3 College of Bioscience and Biotechnology, Hunan Agricultural University, Changsha, Hunan, China; 4 Institute of Forestry Sciences, Guangxi Dongmen Forest Farm, Fusui, Guangxi, China; Beijing Normal University, CHINA

## Abstract

Nitrogen (N) fertilization is necessary to sustain productivity in eucalypt plantations, but it can increase the risk of greenhouse gas emissions. However, the response of soil greenhouse gas emissions to N fertilization might be influenced by soil characteristics, which is of great significance for accurately assessing greenhouse gas budgets and scientific fertilization in plantations. We conducted a two-year N fertilization experiment (control [CK], low N [LN], middle N [MN] and high N [HN] fertilization) in two eucalypt plantations with different soil characteristics (higher and lower soil organic carbon sites [HSOC and LSOC]) in Guangxi, China, and assessed soil–atmosphere greenhouse gas exchanges. The annual mean fluxes of soil CO_2_, CH_4_, and N_2_O were separately 153–266 mg m^-2^ h^-1^, -55 –-40 μg m^-2^ h^-1^, and 11–95 μg m^-2^ h^-1^, with CO_2_ and N_2_O emissions showing significant seasonal variations. N fertilization significantly increased soil CO_2_ and N_2_O emissions and decreased CH_4_ uptake at both sites. There were significant interactions of N fertilization and SOC level on soil CO_2_ and N_2_O emissions. At the LSOC site, the annual mean flux of soil CO_2_ emission was only significantly higher than the CK treatment in the HN treatment, but, at the HSOC site, the annual mean flux of soil CO_2_ emission was significantly higher for both the LN (or MN) and HN treatments in comparison to the CK treatment. Under the CK and LN treatments, the annual mean flux of N_2_O emission was not significantly different between HSOC and LSOC sites, but under the HN treatment, it was significantly higher in the HSOC site than in the LSOC site. Correlation analysis showed that changes in soil CO_2_ and N_2_O emissions were significantly related to soil dissolved organic carbon, ammonia, nitrate and pH. Our results suggested significant interactions of N fertilization and soil characteristics existed in soil–atmosphere greenhouse gas exchanges, which should be considered in assessing greenhouse gas budgets and scientific fertilization strategies in eucalypt plantations.

## Introduction

Forest plantations with fast growing tree species have quickly expanded in recent years to meet the increasing demand for timber and timber products [1]. Eucalypt, an important afforestation tree species, has been introduced to many tropical or subtropical regions [2]. In southern China, eucalypt plantations covered about 3,680,000 hm^2^ in 2010 [3]. Nitrogen (N) fertilization is necessary to sustain plantation productivity [4], but it also increases the risk of greenhouse gas emissions [5]. The greenhouse gas emissions have been extensively studied in crop lands and forests [6–9]; however, greenhouse gas fluxes in introduced eucalypt plantations and their responses to N fertilization have been seldom reported in southern China [10]. This limits our understanding of the effects of eucalypt planting on greenhouse gas emissions in this area.

The effects of N fertilization on soil–atmosphere exchanges of greenhouse gas have been extensively studied, but the results have not been consistent, varying from positive to negative in different studies [11–18] or even in the same study [19,20]. These variations impeded accurate assessment of global greenhouse gas fluxes [21]. It has been reported that the response of soil greenhouse gas fluxes to N addition depends on the N status of ecosystems [15], or is influenced by soil properties, such as dissolved organic matter or ratios of N to other nutrients [17,22,23]. Soil organic carbon (SOC) is a very important soil property and might influence responses of soil greenhouse gas fluxes to N addition through coupling between carbon and N cycles [24,25]. However, the interactions of N fertilization and SOC on soil greenhouse gas fluxes have rarely been studied.

In order to assess soil greenhouse gas fluxes in eucalypt plantations and investigate the interactive effects of N fertilization and SOC level on soil greenhouse gas fluxes, two eucalypt plantations in southern China with different SOC contents were selected as study sites (higher and lower soil organic carbon sites [HSOC and LSOC]), and four N fertilization treatments [no fertilization control (CK), low (LN), middle (MN) and high (HN) level N fertilization] were applied in each plantation. The soil–atmosphere exchanges of greenhouse gas were measured using static chambers and chromatography during May 2013 to April 2015. The early results of the first growing season had been published as communications in Chinese [26,27]. We hypothesized that (1) N fertilization would increase soil CO_2_ and N_2_O emissions and decrease CH_4_ uptake, and (2) the responses of soil–atmosphere exchanges of greenhouse gas to N fertilization would be larger in HSOC site than in LSOC site.

## Materials and methods

### Site description

This study was conducted in the Dongmen Forest Farm (with the permission of Guangxi Dongmen Forest Farm) (22°16′–22°30′N, 107°13′–107°59′E), which is located in Dongmen county, Chongzuo city, Guangxi province, southern China, with an altitude varying from 140 to 250 masl and an area of 22,000 hm^2^ on hilly terrain. This region is characterized by a typical subtropical monsoon climate, with mean annual temperatures of 21.2–22.3°C and annual rainfall of 1100–1300 mm. Soils in the region are mainly lateritic red soils derived from arenaceous shale with a profile depth of about 80 cm. Soils’ pH range from 4 to 5 [3].

Eucalypt (*Eucalyptus urophylla* × *grandis*) is the dominant tree species planted in the Dongmen Forest Farm and Guangxi province. In the eucalypt plantations, tree density was about 1400 trees hm^-2^ and the rotation was 5 years. Prior to eucalypt planting, clear-cutting, fire clearance and reclamation were completed. Then, a base fertilizer (500 g/seedling, N:P:K = 10:15:5) was placed into a 10-cm-deep soil hole and covered with soil. Topdressing was performed once a year during the first 3 years after eucalypt planting (1^st^ year, 250 g/seedling; 2^nd^ year, 500 g/seedling; 3^rd^ year, 500 g/seedling; N:P:K = 10:15:5). The application of herbicide (glyphosate) was performed once a year during the first 3 years after planting and consequently the coverage of understory plants was less than 50%. The understory plants were dominated by *Eupatorium odoratum*, *Rhodomyrtus tomentos* and *Miscanthus floridulus*. The leaf, branch and bark litter were kept in the plantation during the plant growth period, but at harvest time most branch litter was removed and burned.

### Experimental design

The aim of this paper was to study the impacts of N fertilization on soil–atmosphere greenhouse gas exchanges in eucalypt plantations with different SOC contents. In order to select two eucalypt plantations with significantly different SOC contents within the Dongmen Forest Farm, soils from 20 eucalypt plantations (1–2 years in age) with similar above ground conditions were collected for physical and chemical analysis. The eucalypt plantation with the highest SOC was selected as the HSOC site and that with the lowest SOC was selected as the LSOC site. The land use histories of the two eucalypt plantations were similar (each represented the second rotation of eucalypt planting after conversion from *Pinus* to *Eucalyptus*). The eucalypt in both study sites were planted during May to October 2012 and the tree density was about 1400 tree hm^-2^. The tree height (TH), diameter at breast height (DBH) and their increments during May, 2013 to May, 2014 were described in Table 1. Understory coverage was less than 50% due to herbicide application. The initial soil properties were described in Table 2.

**Table 1:** Tree growth under different N treatments in eucalypt plantations with different soil organic carbon levels from 2013 to 2014.

Site	N treatment	May, 2013		May, 2014		Increment	
		DBH cm	TH m	DBH cm	TH m	DBH cm	TH m
LSOC	CK	6.47±0.29	7.15±0.30	10.05±0.29	10.67±0.30	3.58±0.06	3.52±0.05
	LN	6.43±0.56	6.95±0.43	10.06±0.52	10.99±0.58	3.63±0.16	4.04±0.56
	MN	6.68±0.07	7.53±0.23	10.34±0.08	11.73±0.31	3.66±0.02	4.20±0.45
	HN	6.48±0.08	7.10±0.16	10.30±0.33	11.63±0.51	3.82±0.28	4.53±0.55
HSOC	CK	4.77±0.06	5.42±0.12	8.49±0.41	10.02±0.36	3.72±0.37	4.60±0.25
	LN	4.94±0.26	5.65±0.32	8.88±0.27	10.39±0.43	3.93±0.19	4.74±0.13
	MN	5.12±0.20	5.76±0.17	9.19±0.21	10.74±0.26	4.06±0.07	4.98±0.11
	HN	5.39±0.19	5.70±0.16	9.62±0.20	10.94±0.30	4.23±0.03	5.24±0.13

LSOC and HSOC, study sites with lower and higher soil organic carbon levels. CK, LN, MN and HN, control and low, middle, high nitrogen fertilization treatments. DBH, diameter at breast height; TH, tree height.

**Table 2:** Initial soil properties of LSOC and HSOC sites in the eucalypt plantations.

Site	Bulk Density g cm^-3^	Soil mechanical composition			pH	SOC g kg^-1^	TN g·kg^-1^	DOC mg kg^-1^	NH_4_^+^-N mg·kg^-1^	NO_3_^-^-N mg·kg^-1^	AP mg·kg^-1^	AK mg·kg^-1^
		Clay %	Silt %	Sand %								
LSOC	1.13±0.07	64	32	4	3.99±0.02	19.9±0.6 b	1.3±0.1 b	441±19	9.68±0.90	8.44±1.93	1.93±0.22	37.0±4.0 b
HSOC	1.18±0.05	62	33	5	3.91±0.04	24.6±1.6 a	1.5±0.2 a	433±23	11.24±1.11	6.80±0.42	1.91±0.17	58.2±3.2 a

LSOC and HSOC, study sites with lower and higher soil organic carbon levels. SOC, soil organic carbon; TN, total nitrogen; DOC, dissolved organic carbon; NH_4_^+^-N, ammonia nitrogen; NO_3_^-^-N, nitrate nitrogen; AP, available phosphorus; AK, available potassium. Soil properties with different letters are significantly different at *p* < 0.05 level.

At each site, 12 plots (10 m × 10 m) were separated by a 5 m wide buffer strip. High, middle, and low level N fertilization treatments (HN: 334 kg N·hm^-2^, MN: 167 kg N·hm^-2^ and LN: 84 kg N·hm^-2^) and a no fertilization control (CK: 0 kg·hm^-2^), with three replications, were randomly arranged in the 12 plots. Urea formaldehyde was used in this experiment as N fertilizer to simulate the controlled release of fertilizer used in the Dongmen Forest Farm. On May 19–20, 2013 and May 25–30, 2014, urea formaldehyde was fertilized in soil holes (about 10 cm depth) that were about 30 cm away from each tree and covered with soil.

**Fig 1:**
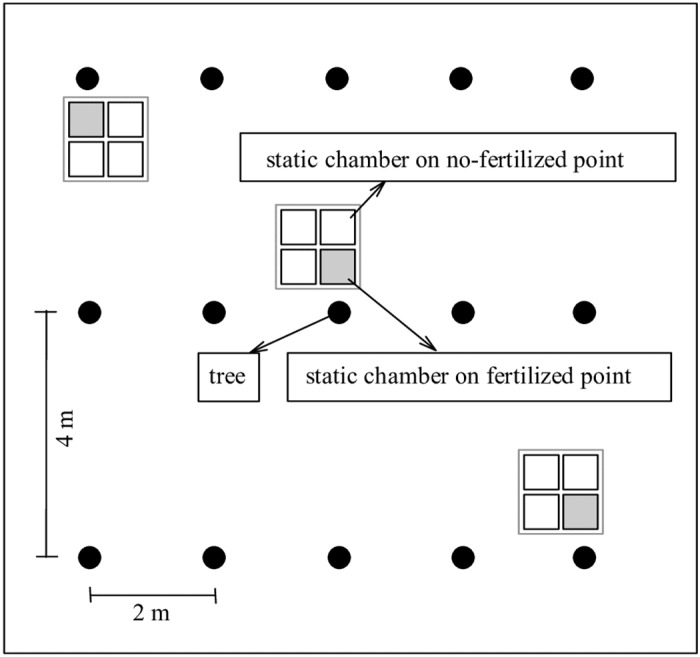
Greenhouse gas sampling points at tree and plot level in eucalypt plantations.

In each plot, three trees aligned in a diagonal pattern were selected. For each tree, gas sampling was performed at four points, a fertilized point and three non-fertilized points (Fig 1). The tree-level soil greenhouse gas fluxes were calculated by averaging the fluxes of the four points and the plot-level fluxes were calculated by averaging the three tree-level fluxes at each plot.

### Greenhouse gas flux measurements

Soil–atmosphere exchange of CO_2_, CH_4_, and N_2_O was measured using a static chamber technique and gas chromatography. The static chamber consisted of two parts, a stainless-steel base box (without top and bottom, but with a 4 cm deep × 4 cm wide trough on top of the side wall, length × width × height = 0.4 m × 0.4 m × 0.1 m) and a removable transparent acrylic cover box (without bottom, length × width × height = 0.4 m × 0.4 m × 0.4 m). At the gas sampling location, the base box was permanently inserted directly into the soil about 5 cm, and the cover box was placed on top of the base box and sealed by filling water into the trough during sampling and removed afterwards. On the side wall of each chamber, a fan (10 cm in diameter) was installed to mix the air during sampling and a tube was installed to balance air pressure between the inside and outside of the chamber. The sampling tube was installed on the top wall of each chamber for gas sampling. Gas samples (300 ml) were collected in 500 mL gas sampling bags (Dalian Hede Technologies LTD., Dalian, China) using a gas sampler QC-1 (Beijing Municipal Institute of Labour Protection, Beijing, China) at 0, 10, 20, and 30 min after chamber closure.

Gas samples were collected between 09:00 and 11:00 once per month for laboratory analysis and calculation of the greenhouse gas fluxes. Samples were analyzed for CO_2_, CH_4_, and N_2_O concentrations using an Agilent 7890A gas chromatograph (Agilent, Wilmington, DE, USA). The gas fluxes were calculated from the changes in gas concentration in relation to time after chamber closure. The calculation was conducted using the following equation [26]:

F=ρ×(V/A)×(Δc/Δt)×273/(273+T)

Where *F* is gas flux (mg m^-2^ h^-1^ for CO_2_ and μg m^-2^ h^-1^ for CH_4_ and N_2_O), *ρ* is gas density under normal conditions (mg m^-3^), *V* is the volume of the static chamber (m^3^), *A* is the area that the static chamber covered, Δ*c*/Δ*t* is changes in gas concentration (Δ*c*) during a certain time (Δ*t*), and *T* is air temperature (°C).

### Measurement of environmental and soil factors

**Fig 2:**
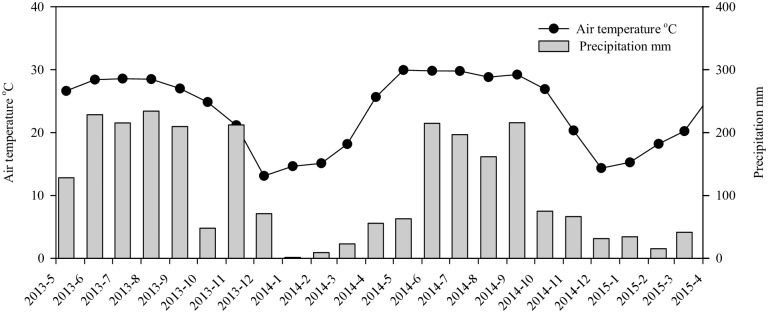
Air temperature and precipitation at Dongmen Forest Farm, Guangxi, China.

Air temperature and precipitation in the Dongmen Forest Farm (Fig 2) were monitored using a rainfall recorder L99-YLWS (Shanghai Fotel LTD., Shanghai, China).

In August 2013 and 2014, 0–20 cm surface soils near the gas sampling points were sampled. Soil pH, dissolved organic carbon (DOC), ammonia N (NH_4_^+^-N), and nitrate N (NO_3_^-^-N) were measured. To measure soil pH, 10 g of air-dried soil were combined with 25 ml of deionized water. The slurry was swirled gently by hand and allowed to settle for 30 min. A Delta 320 pH meter [Mettler-Toledo Instruments (Shanghai) Co., Ltd., Shanghai, China] was used to measure pH of the supernatant. To measure DOC, the soil was extracted with a 0.5 M K_2_SO_4_ solution (soil:solution ratio of 1:5) by shaking for 30 min and the extracts were filtered through filter paper (45 μm). The filtrates were analyzed using a TOC analyzer (Liqui TOC, Elementar, Hanau, Germany). To measure NH_4_^+^-N and NO_3_^-^-N, the soil was extracted with a 2 M KCl solution (soil:solution ratio of 1:5) by shaking for 30 min and the extracts were filtered through filter paper (45 μm). The filtrates were analyzed using a continuous flow analyzer (San++, SKALAR, Breda, Netherlands).

### Statistical analysis

An independent-samples t test was used to compare initial soil physical and chemical properties between the HSOC and LSOC sites. Two-way analysis of variance (ANOVA) was used to analyze the impact of N fertilization, SOC, and their interaction on soil properties (August 2014 and August 2015) and greenhouse gas exchanges (annual mean fluxes during May 2013 to April 2014 and during May 2014 to April 2015). When significant F tests were obtained, Tukey’s test was used to identify the significance of differences between N treatments in the HSOC and LSOC sites. The relationship between soil greenhouse gas fluxes and soil properties were analyzed using Pearson correlation analysis. All statistical analyses were performed using SPSS 16.0 for windows (SPSS Inc., Chicago, IL, USA).

## Results

### Annual mean fluxes and seasonal variations of greenhouse gas exchanges in eucalypt plantations

**Fig 3:**
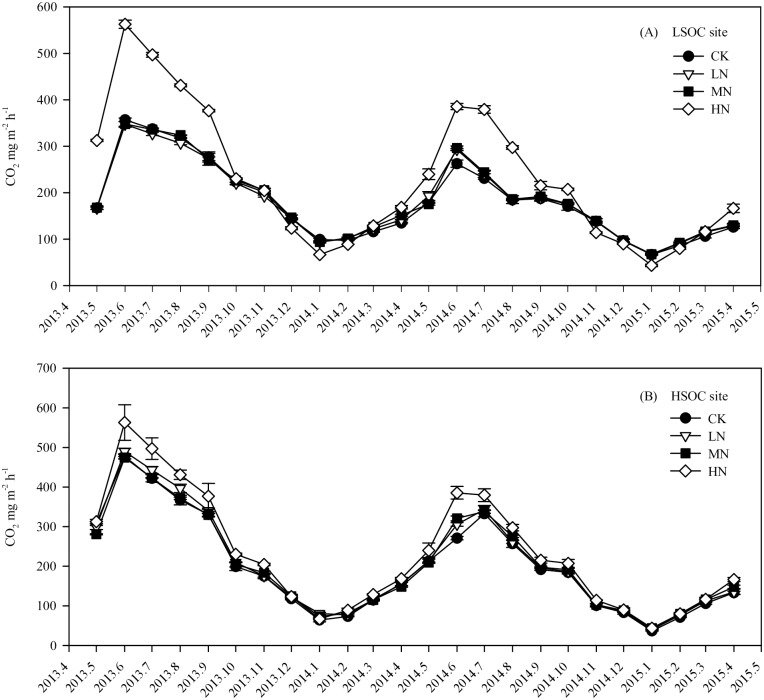
Monthly soil CO_2_ flux in eucalypt plantations with different SOC contents and N fertilization treatments. LSOC and HSOC, study sites with lower and higher soil organic carbon levels. CK, LN, MN and HN, control and low, middle, high nitrogen fertilization treatments.

**Fig 4:**
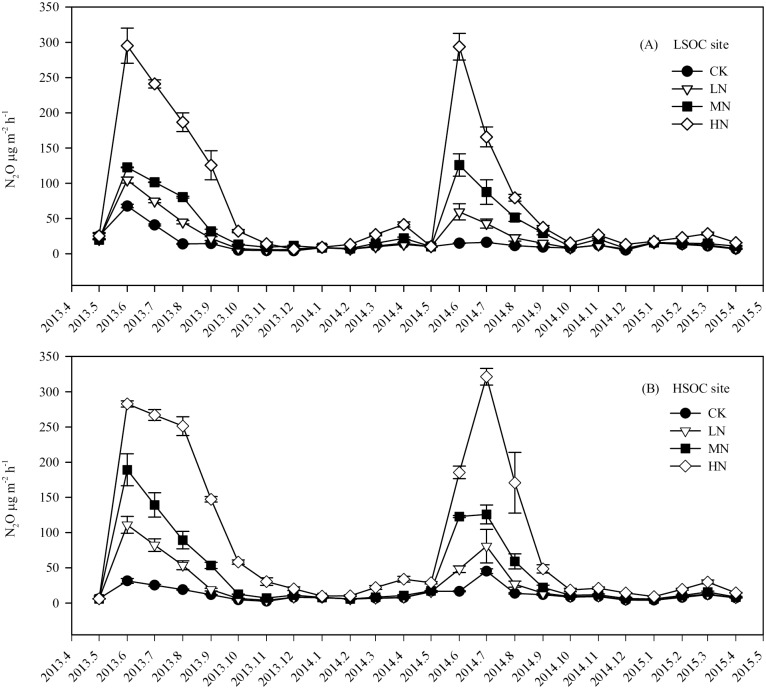
Monthly soil N_2_O fluxes in eucalypt plantations with different SOC contents and N fertilization treatments. LSOC and HSOC, study sites with lower and higher soil organic carbon levels. CK, LN, MN and HN, control and low, middle, high nitrogen fertilization treatments.

**Fig 5:**
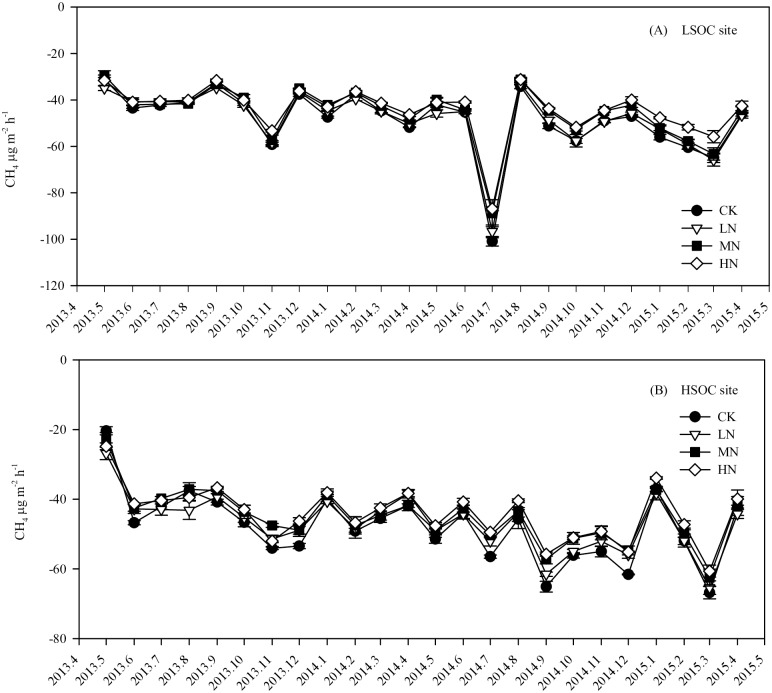
Monthly soil CH_4_ fluxes in eucalypt plantations with different SOC contents and N fertilization treatments. LSOC and HSOC, study sites with lower and higher soil organic carbon levels. CK, LN, MN and HN, control and low, middle, high nitrogen fertilization treatments.

During our experiment, the annual mean fluxes of soil CO_2_, CH_4_, and N_2_O were separately 153–266 mg m^-2^ h^-1^, -55 –-40 μg m^-2^ h^-1^, and 11–95 μg m^-2^ h^-1^ under different N fertilization treatments at both sites. In the plantations with no N fertilization, soil CO_2_ and N_2_O emissions showed significant seasonal variations, which were higher in warm and wet months (June–September) and lower in cold and dry months (December–March) (Figs 3 and 4), but soil CH_4_ uptake did not show significant seasonal variation (Fig 5).

### Effects of nitrogen fertilization on soil greenhouse gas exchanges

**Fig 6:**
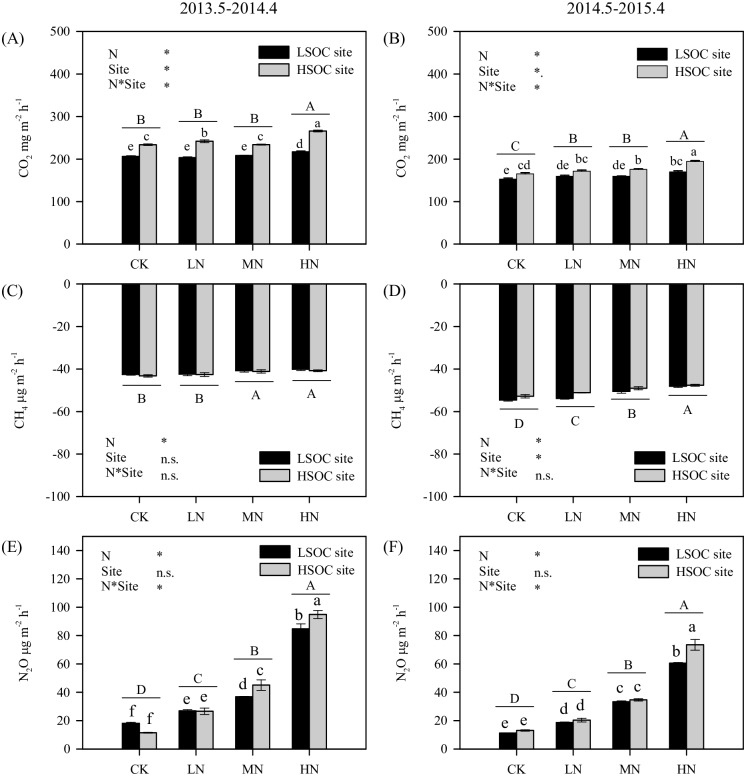
Annual mean soil CO_2_, CH_4_, and N_2_O fluxes in eucalypt plantations with different SOC contents and N fertilization treatments. LSOC and HSOC, study sites with lower and higher soil organic carbon levels. CK, LN, MN and HN, control and low, middle, high nitrogen fertilization treatments. Greenhouse gas fluxes with different letters (lower-case letters, comparison between different N fertilization treatments in different sites; capital letters, comparison between different N fertilization treatments) are significantly different at *p* < 0.05 level.

N fertilization significantly increased soil annual mean fluxes of CO_2_ and N_2_O emissions, and decreased soil CH_4_ uptake in eucalypt plantations (*p*<0.05) (Fig 6). During 2013–2014, the annual mean flux of soil CO_2_ emission was significantly higher under the HN treatment (217–266 mg m^-2^ h^-1^) than under the CK treatment (206–234 mg m^-2^ h^-1^). During 2014–2015, the annual mean flux of soil CO_2_ emissions was significantly higher under LN, MN, and HN treatments (159–195 mg m^-2^ h^-1^) than under the CK treatment (153–165 mg m^-2^ h^-1^). During 2013–2014, the annual mean fluxes of soil CH_4_ uptake were significantly lower under the MN and HN treatments (-4 1– -40 μg m^-2^ h^-1^) than under the CK and LN treatments (-43 –-42 μg m^-2^ h^-1^). In 2014–2015, the annual mean fluxes of soil CH_4_ uptake decreased with the amount of added N, with significant differences between different treatments (*p*<0.05). In both years, the annual mean flux of soil N_2_O emission (11–95 μg m^-2^ h^-1^) increased with the amount of added N, with significant differences between different treatments (*p*<0.05).

### Differences in nitrogen-induced greenhouse gas exchanges between the two study sites

The responses of soil CO_2_ and N_2_O emissions to N fertilization were significantly different between HSOC and LSOC sites. Two-way ANOVA showed significant interactions of N fertilization and SOC level on soil CO_2_ and N_2_O emissions. At the LSOC site, the annual mean soil CO_2_ flux was only significantly higher under the HN treatment in comparison to the CK treatment. However, at the HSOC site, soil CO_2_ fluxes were significantly higher under LN and HN treatments (2013–2014) or under MN and HN treatments (2014–2015) than under the CK treatment (Fig 6a and 6b). Under the CK and LN treatments (2013–2014) or under the CK, LN, and MN treatments (2014–2015), the annual mean flux of N_2_O was not significantly different between HSOC and LSOC sites. However, under MN and HN (2013–2014) or under the HN treatment (2014–2015), the annual mean flux of N_2_O was significantly higher in the HSOC site than in the LSOC site (Fig 6e and 6f).

### Soil properties and their relationship with greenhouse gas exchanges

In August 2013, after 3 months of N fertilization, soil DOC, NH_4_^+^-N and NO_3_^-^-N were measured (Table 3). Soil DOC concentrations showed an increasing trend with the amount of fertilized N, but the differences were not significant. Soil NH_4_^+^-N concentrations were significantly influenced by N fertilization, SOC level and their interactions. In the LSOC site, soil NH_4_^+^-N concentrations did not show significant differences between N fertilization treatments. However, in the HSOC site, NH_4_^+^-N concentrations were significantly higher under the HN treatment than under the LN treatment. Soil NO_3_^-^-N concentrations were influenced by N fertilization, which was significantly higher under the HN treatment than under the CK and LN treatments at both sites.

**Table 3:** Soil chemical properties in eucalypt plantations with different SOC contents and N fertilization treatments.

Treatment		August, 2013			August, 2014		
Site	N	DOC mg kg^-1^	NH_4_^+^-N mg·kg^-1^	NO_3_^-^-N mg·kg^-1^	pH	NH_4_^+^-N mg·kg^-1^	NO_3_^-^-N mg·kg^-1^
LSOC	CK	407.45±6.93	11.87±0.46 b	4.50±0.35 bc	4.51±0.11	32.14±4.18	8.16±0.42 b
	LN	449.72±28.08	12.08±0.01 b	3.18±0.62 c	4.25±0.02	30.23±2.11	13.47±3.32 ab
	MN	477.90±32.40	12.38±0.76 b	4.50±0.61 bc	4.23±0.09	27.03±0.36	18.77±3.58 ab
	HN	477.21±42.51	11.93±0.45 b	11.68±1.83 a	4.20±0.11	27.46±0.35	23.77±3.02 a
HSOC	CK	464.86±5.72	13.00±0.75 ab	4.23±0.36 c	4.44±0.05	27.55±1.03	7.70±1.20 b
	LN	485.21±38.62	11.43±0.42 b	4.02±1.38 c	4.38±0.01	27.27±5.48	10.45±2.42 b
	MN	508.17±27.12	13.18±0.10 ab	4.55±0.77 bc	4.34±0.06	33.79±4.20	10.14±1.48 b
	HN	509.21±3.62	16.65±1.73 a	10.40±2.22 ab	4.27±0.07	29.28±1.24	12.41±1.03 ab

LSOC and HSOC, study sites with lower and higher soil organic carbon levels. CK, LN, MN and HN, control and low, middle, high nitrogen fertilization treatments. DOC, dissolved organic carbon; NH_4_^+^-N, ammonia nitrogen; NO_3_^-^-N, nitrate nitrogen. Soil properties with different letters are significantly different at *p* < 0.05 level.

In August 2014, soil pH, NH_4_^+^-N, and NO_3_^-^-N were measured (Table 3). Soil pH showed a decreasing trend with the amount of fertilized N, but the differences were not significant. Soil NH_4_^+^-N concentrations showed no significant differences between different N fertilization treatments at both sites. Soil NO_3_^-^-N concentrations showed an increasing trend with the amount of fertilized N.

Pearson correlation showed that soil CO_2_ flux positively correlated with soil DOC and NH_4_^+^-N concentrations (*p*<0.05) in August 2013 and CH_4_ flux positively correlated with NO_3_^-^-N concentrations (*p*<0.01) in August 2014. Soil N_2_O flux positively correlated with NH_4_^+^-N and NO_3_^-^-N concentrations (*p*<0.01) in August 2013, and negatively correlated with soil pH (*p*<0.05) in August 2014 (Table 4).

**Table 4:** Correlation analysis of soil greenhouse gas fluxes and chemical properties in eucalypt plantations.

	Aug, 2013			Aug, 2014		
	DOC	NH_4_^+^-N	NO_3_^-^-N	pH	NH_4_^+^-N	NO_3_^-^-N
CO_2_	0.42*	0.57**	0.25	0.11	0.03	-0.35
CH_4_	-0.20	0.33	0.16	-0.39	0.08	0.55**
N_2_O	0.40	0.54**	0.79**	-0.46*	-0.05	0.25

DOC, dissolved organic carbon; NH_4_^+^-N, ammonia nitrogen; NO_3_^-^-N, nitrate nitrogen.

*, the effect is significant at *p* < 0.05 level.

**, the effect is significant at *p* < 0.01 level.

## Discussion

The annual mean soil-atmosphere greenhouse gas fluxes in our study fall in the range of greenhouse gas fluxes reported by previous studies [19,20,28–30].

Our result suggested that N fertilization stimulated soil CO_2_ emissions (Fig 6a and 6b). N fertilization might stimulate soil CO_2_ emissions through two pathways. The first way is by stimulating light organic carbon decomposition and increasing soil DOC concentration (Table 3), resulting in higher soil heterotrophic respiration and higher soil CO_2_ emission fluxes. The second way is by stimulating plant root growth and activity, resulting in higher autotrophic respiration [4,31]. This was not tested in this study due to lack of root data.

A higher stimulative effect of N fertilization on soil CO_2_ emissions was found at the HSOC site. On the one hand, the higher SOC generally related to higher soil available carbon, microbial biomass, and microbial activities [32], which would stimulate soil respiration [33,34] and increase soil CO_2_ emissions in response to N addition. In this study, soil DOC concentrations were 407–477 mg kg^-1^ in the LSOC site and 465–509 mg kg^-1^ in the HSOC site, which might explain the higher priming effect of N fertilization on soil CO_2_ emission in HSOC site. On the other hand, N fertilization could inhibit soil respiration by decreasing soil pH [35]. Our results showed that soil pH significantly decreased with increases in fertilized N (Table 3), which likely reduced soil respiration and counteracted the stimulation effect of N fertilization. Changes in soil pH induced by N fertilization could be influenced by soil cation exchange capacity, which usually correlated with SOC content with positive relationship [36,37]. With increases in fertilized N, soil pH decreased from 4.51 to 4.20 at the LSOC site and from 4.44 to 4.27 at the HSOC site, suggesting that soils with higher organic carbon could buffer N-induced decreases of soil pH. Thus, the significantly higher soil CO_2_ emission fluxes under the HN treatment at the HSOC site might be the result of higher soil DOC concentrations and smaller decreases in soil pH.

N fertilization significantly decreased CH_4_ uptake, which was consistent with previous studies [12,23]. The inhibition of N fertilization on soil CH_4_ uptake might be caused by competitive inhibition of CH_4_ monooxygenase [38], toxic inhibition by hydroxylamine and nitrite produced during nitrification process [39], high osmotic pressure caused by high N ammonia and nitrate concentration [40], and toxicity caused by decreased soil pH [41]. We found that soil NO_3_^-^-N concentration increased and soil pH decreased with increases in fertilized N (Table 3), which explained the decreased soil CH_4_ uptake in the MN and HN treatments.

N fertilization significantly increased soil N_2_O emission fluxes, which might be explained by higher N availability caused by N fertilization (Table 3). That result was consistent with Zhang et al.[19], who studied the responses of N_2_O emissions to simulated N deposition in three tropical forests in southern China and found that N fertilization increased soil N_2_O emissions through increasing N availability and stimulation of nitrification and denitrification processes.

The increase of soil N_2_O emission fluxes caused by N fertilization was significantly higher at the HSOC site (from 12 μg m^-2^ h^-1^ to 95 μg m^-2^ h^-1^) than at the LSOC site (from 18 μg m^-2^ h^-1^ to 85 μg m^-2^ h^-1^) (Fig 6e and 6f). That might be explained by differences in soil carbon availability between the two sites. Denitrification, an important soil process producing N_2_O, is generally restricted by substrate availability, energy sources, and presence of anaerobic environments. The higher DOC concentrations at the HSOC site could offer more energy and electron acceptors for denitrification [42], enlarging the soil N_2_O emission responses to N fertilization. Additionally, higher respiration at the HSOC site might exhaust soil O_2_ and create an anaerobic environment, which would be suitable for denitrification and N_2_O production [43].

Our results showed that the responses of soil–atmosphere CO_2_ and N_2_O exchanges to N fertilization were different at the LSOC and HSOC sites. So, when assessing N-induced soil greenhouse gas emissions, soil characteristics, such as SOC, should be considered in attempt to reduce the variation of emission factors [21]. Higher SOC is generally related to higher soil fertility and plant productivity [44], but it also stimulates the responses of soil CO_2_ and N_2_O emissions to N fertilization. Thus, in order to reduce the risk of N-induced greenhouse emissions, N fertilizer should be applied according to soil properties.

## Conclusion

Our study found that the annual mean fluxes of soil CO_2_, CH_4_, and N_2_O in eucalypt plantations in Guangxi, China were separately 153–266 mg m^-1^ h^-1^, -55 –-40 μg m^-1^ h^-1^ and 11–95 μg m^-2^ h^-1^. N fertilization significantly increased soil CO_2_ and N_2_O emissions and decreased soil CH_4_ uptake. The stimulative effects of N fertilization on soil CO_2_ and N_2_O emissions were significantly higher at the HSOC site than at the LSOC site. Thus, the interaction of N fertilization and soil characteristics should be considered in assessing greenhouse gas budgets and scientific fertilization in eucalypt plantations.
